# Lymphoepithelioma-Like Carcinoma of the Salivary Gland: Is Radiotherapy Alone Adequate?

**DOI:** 10.1155/2011/618650

**Published:** 2011-11-02

**Authors:** Orit Kaidar-Person, Abraham Kuten, Salem Billan

**Affiliations:** Division of Oncology, Rambam Health Care Campus, P.O. Box 9602, Haifa 31096, Israel

## Abstract

*Introduction*. Lymphoepithelioma-like carcinoma (LELC) of the salivary gland is a rare tumor. Currently, surgery with or without radiotherapy is the recommended treatment for all salivary gland carcinomas. However, in contrast to other high-grade salivary gland carcinomas, LELCs are considered radiosensitive. There are only a few published reports of radiotherapy alone for the treatment of salivary gland LELC. *Case*. We present two cases of LELC of the salivary gland. One was treated with surgery and postoperative radiotherapy, and the other was given a single cycle of chemotherapy and then radiotherapy. Currently, both patients have no evidence of disease. *Conclusion*. Radiotherapy as a single modality should be reevaluated. The role of systemic chemotherapy to gain systemic control should be addressed due to noteworthy metastatic disease.

## 1. Introduction

Lymphoepithelioma is a term that was used by Regaud and Schmincke in 1921 to describe certain tumors of the nasopharynx [1]. Histologically, the tumor is characterized by undifferentiated nonkeratinizing squamous-cell carcinoma, featuring syncytial cytoplasm, vesicular nuclei, and large central nucleoli with a remarkably inflammatory background (lymphoid stroma) seen as cell clusters or single cells [1–3]. Significant correlation has been reported between this tumor type and the Epstein Barr virus (EBV) [2–5] and, more recently, with the human papilloma virus (HPV) [3] as well. Carcinomas with similar morphologic features have rarely been described in other organs and are referred to as lymphoepithelioma-like carcinoma (LELC). These have primarily been observed in the upper aerodigestive tract, including the salivary glands (mainly parotid), oral cavity, and tonsils, but have also been found in the stomach, thyroid, bladder, ureters, uterine cervix, ovaries, skin, and other anatomic locations [4–9]. These tumors comprise a significant proportion of salivary gland carcinomas seen in Chinese and Eskimo populations [5, 10].

The mainstay of treatment for salivary gland tumors is surgical resection. Postoperative radiotherapy is indicated for unfavorable prognostic features, such as close or positive surgical margins, high-grade malignancy, perineural invasion, tumor infiltration of adjacent organs, recurrent disease, and regional metastasis, and has been shown to improve local-regional control and survival, particularly in advanced-stage disease [11]. In contrast to other high-grade salivary gland carcinomas, LELCs are considered radiosensitive [1, 12], but, due to their rarity, the optimal treatment for salivary gland LELC is not well established and relies on limited retrospective reports. Radiotherapy as a single modality for the treatment of LELC was previously reported by Dubey et al. [12]. In this paper we review two cases of LELC of the salivary gland that were treated with different treatment modalities and discuss related literature.

## 2. First Case

A 19-year-old female with no history of chronic diseases complained of a swelling in the floor of her mouth. Physical examination revealed a 5-6 cm right sublingual swelling. Biopsy from the sublingual lesion was consistent with LELCs, most probably a primary tumor of the sublingual salivary gland, somewhat similar to those of nasopharyngeal origin. No distant metastases were noted. The patient was referred to the Maxillofacial Surgery Department and underwent a resection of the sublingual salivary gland, a marginal mandibulectomy from teeth 33 to 47, partial glossectomy and bilateral cervical dissection, reconstruction of the jaw with titanium plate, prosthetic dental implants, and a myocutaneous free-flap from her left arm. The pathology report indicated LELC with a maximal diameter of 3 cm. The tumor invaded the fat and mucosa with 0.3 cm clear lateral margins and 0.5 cm clear caudal margins. The posterior margins were microscopically involved by the tumor. No lymph node metastases were noted.

A week after the surgery, the patient suffered from necrosis of the flap and it was excised. During this procedure, posterior extension of the resection was performed due to positive microscopic margins. The wound was primarily closed. The pathology report showed no residual disease of the new resected specimen. A month after surgery, the patient suffered from a cervicocutaneous fistula that was treated conservatively. She underwent a percutaneous endoscopic gastrostomy and was referred to the Oncology Department for radiotherapy. She received external beam irradiation to the neck and surgical bed, with a total dose of 50 Gy to the bilateral neck and the tumor bed and a boost dose of 16 Gy to the tumor bed. Two and a half years after the diagnosis, the patient has no evidence of disease, although she suffers from severe neck fibrosis.

## 3. Second Case

A 62-year-old female was diagnosed with a mass in the left parotid gland and left supraclavicular lymphadenopathy. A biopsy from both masses revealed LELC. Random biopsies from the nasopharynx were negative for malignancy. PET-FDG scan showed pathological uptake in the left parotid gland and left supraclavicular lymph nodes. The patient was given a cycle of chemotherapy with docetaxel (120 mg intravenous), cisplatin (130 mg intravenous), and 5-fluorouracil (continuous drip of 1300 mg/per 22 hours for 5 days). During the treatment, the patient suffered from grade 2 nausea and vomiting. A few days after the first chemotherapy cycle, she was hospitalized for 6 days due to neutropenia and fever. She received antibiotics and daily injections of granulocyte colony-stimulating factor (GCSF). It was decided not to continue chemotherapy, and the patient was referred for radiotherapy. Prior to radiation, she underwent a percutaneous endoscopic gastrostomy. The patient received external beam irradiation; the irradiation field included bilateral neck and left parotid with a total dose of 50 Gy and a boost of 16 Gy to the left parotid gland and involved the left supraclavicular lymph nodes. PET-FDG scan performed three months after the end of the radiation treatment showed no pathological uptakes. Four years after the initial diagnosis of LELC, the patient has no evidence of disease. It is noteworthy that this patient did not undergo surgical treatment of the tumor.

## 4. Discussion

Salivary gland malignancies are uncommon and comprise approximately 3% of annual, newly diagnosed head and neck carcinomas in North America [13, 14]. Of these, LELCs comprise a relatively rare entity, which is more common in specific populations such as Eskimos and Chinese. In his paper in 1929, Ewing [1] noted that LELC tumors occur at all ages, but are especially frequent between 30 and 60 years of age. Dubey et al. [12] reported a range of 30–80 years, with a median age of 60 and a male predominance. Typically, the first symptom is an enlargement of the cervical lymph nodes [1, 12], as seen in one of the cases described in this paper, making surgery a difficult task with extensive lymph node dissection. Furthermore, regional lymph node involvement may indicate the presence of distant metastases [1, 12].

The prognosis of salivary gland tumors depends on the histological type, stage, grade, completeness of the surgical resection, and, when the resection is incomplete, whether or not postoperative radiotherapy is administered [11, 13]. The N-stage and the histological type (LELC/acinic cell carcinoma, adenocystic carcinoma, or others) were the only significant prognostic factors that governed overall survival [13]. For all histological types, the N-stage was the only significant factor that determined the rate of distant metastasis and the rate of locoregional failure. The generally poor outcome of the treatment of salivary gland carcinomas with irradiation alone in several series may be attributed to the use of primary radiotherapy for patients with locally advanced lesions or distant metastases at presentation [11, 14–18]. Moreover, in contrast to other high-grade salivary gland carcinomas, LELCs are considered to be radiosensitive [1, 12, 13, 19, 20]. This was also demonstrated in cases of oropharynx and nasopharyngeal LELCs, in which good local control rates were achieved by radiotherapy alone [21–23].

Dubey et al. [12] retrospectively reviewed cases of LELC of the head and neck where the primary tumor was located outside the nasopharynx. Of 34 patients, only four had tumors originating in the salivary gland: two of the parotid gland and two of the submandibular gland. Three different treatment modalities were applied: 24 patients were treated with radiation therapy only (including all the patients with salivary gland origin), two of whom had neck dissections after radiation therapy because of residual lymph node masses. Seven patients underwent excision of the primary tumor followed by radiotherapy. The remaining three patients, two of whom had primary tumors in the supraglottic larynx and one of whom had the primary tumor in the hypopharynx, were treated with surgery only (laryngectomy, partial pharyngectomy, and neck dissection); one of these patients was also given adjuvant chemotherapy. For patients who received radiotherapy, the median radiation dose given to the primary tumor was 65 Gy (range, 46–78 Gy), similar to the radiation dose we used in both our cases. A variety of radiotherapy modalities were used including cobalt 60, 6 MV photons, a mixture of photons, and neutrons and orthovoltage X-rays; this may explain the occurrence of severe acute mucositis in three patients which resulted in treatment interruptions (7–21 days).

With the current use of advanced radiotherapy techniques, such as IMRT and IGRT, complication rates are expected to be lower. There was no statistical significance in the 5-year local and regional control rates in the all-patients group and in the radiotherapy-alone group. It was recommended that locoregional treatment strategy for patients with non-nasopharyngeal LELC should include radiotherapy to all primary tumors. Because the main cause of treatment failure was distant metastases and patients with regional lymphadenopathy had relatively high rates of distant metastases, it was recommended that patients who present with regional lymphadenopathy should be treated with chemotherapy as well [12]. In the second case we presented, the patient received one cycle of chemotherapy due to cervical lymph node involvement. Interestingly, after one cycle of chemotherapy, the lesion in the left parotid and the lymphadenopathy regressed significantly.

Two studies of salivary gland LELC in which surgery was the main treatment demonstrated interesting results. In the first study, which included seven cases of LELC of parotid gland origin treated by surgery and postoperative radiotherapy [13], two patients experienced relapses. One of these patients had not received elective neck irradiation and isolated regional relapse developed six years after parotidectomy and postoperative radiotherapy of the primary tumor bed. The second patient had local recurrence in the parotid gland despite total parotidectomy with clear surgical margins and postoperative radiotherapy (50 Gy) 3.5 years after the primary treatment implying that, although the tumor is radiosensitive, 50 Gy might be insufficient for microscopic residual disease. The authors indicted that both cases of relapse were successfully salvaged by further treatments; the regional recurrence was treated by performing radical neck dissection and postoperative regional radiotherapy, and the parotid recurrence was treated by a second course of radical radiotherapy (60 Gy) using the 3D conformal method. There were no cases of mortality in this group of patients [13]. The authors did not report the incidence of treatment complications and morbidities, such as facial nerve paralysis, which is a common complication of the treatment of parotid malignancies.

In the second study [24], all eight patients underwent excision of primary salivary gland tumors. Five of these patients with cervical node metastases underwent lymph node dissection. After surgery, seven patients received postoperative radiotherapy with a 60 cobalt machine or 6–10 MV linear accelerator. The total radiation dose to the salivary tumor bed ranged from 39.6 to 67.6 Gy (mean dose, 58.3 Gy; median dose, 59 Gy). Oral mucositis and skin reaction over the radiation field were experienced by each of the seven patients who were irradiated. Major long-term complications included xerostomia (8 patients), neck fibrosis (6 patients), and facial palsy (3 patients). The authors noted that facial palsy was due to tumor encasement of the facial nerve and the surgical procedure. Survival time ranged from 21.4 to 145.2 months. During the study period, there were two cases of mortality, one due to distant metastasis. None of the patients had local or regional failure. Distant metastases to lung, bone, and liver were noted in two patients; the interval between surgery and distant failure was approximately 6.5 months for both [24].

Lymphoepithelioma-like carcinoma of the salivary gland is a rare entity, but, despite the poor differentiation and high-grade histological appearance, its clinical course is better than other poorly differentiated tumors. It is considered to be radiosensitive and has similar histopathologic characteristics as lymphoepithelioma of the nasopharynx [25], in which radiotherapy is the treatment of choice. There are only a few published reports of radiotherapy alone for the treatment of salivary gland LELC with relatively reasonable outcomes. The role of systemic chemotherapy to gain systemic control is not well documented in the literature and should be addressed due to noteworthy metastatic disease. Therefore, surgery followed by radiotherapy, radiotherapy alone, or chemotherapy followed by radiotherapy may be considered as appropriate treatment strategies for LELC of the salivary gland. However, due to the poor functional and cosmetic outcomes of radical surgery, higher toxicity of postoperative radiotherapy compared to radiotherapy alone [26] and comparable outcomes of radiotherapy as a single modality, this treatment strategy should be reevaluated.
